# Treatment of erectile dysfunction by intracavernosal administration of mesenchymal stem cells in patients with diabetes mellitus

**DOI:** 10.1590/S1677-5538.IBJU.2024.0100

**Published:** 2024-04-25

**Authors:** Yerbol Iskakov, Rustam Omarbayev, Rinat Nugumanov, Timur Turgunbayev, Yerkebulan Yermaganbetov

**Affiliations:** 1 Department of Urology JSC “National Scientific Medical Center” Astana Republic of Kazakhstan Department of Urology, JSC “National Scientific Medical Center”, Astana, Republic of Kazakhstan

**Keywords:** Sexual Dysfunctions, Psychological, Erectile Dysfunction, Diabetic Angiopathies

## Abstract

Erectile dysfunction is observed in about 50% of men. It has been found that diabetes mellitus increases its prevalence to 19-86.3%, necessitating attention to a therapeutic strategy. Among the available treatment methods, intracavernosal injections of mesenchymal stem cells have proven to be particularly effective.

**Objective:**

The purpose of study is to assess and analyse the effectiveness of their use in the treatment of erectile dysfunction in patients with diabetes mellitus.

**Materials and Methods:**

The literature search was conducted using systematic methods and analysis in databases such as Web of Science, Scopus, PubMed, Elsevier, and Springer, with 41 sources included for further review.

**Results:**

The study highlights microangiopathic and neuropathic links as key factors in erectile dysfunction development in diabetic patients, stemming from endothelial dysfunction and conductivity disturbances. Mesenchymal stem cell therapy from bone marrow, adipose tissue, and umbilical cord mitigates pathogenic impact through regenerative and anti-apoptotic effects. Due to this, most studies indicate high efficacy of the treatment and rapid therapeutic action through intracavernosal administration. Some studies suggest an increase in the body’s receptor sensitivity to other drugs, such as sildenafil.

**Conclusion:**

From the perspective of further research on this issue, standardising the preparation of stem cells and the treatment method using a large sample size is essential to introduce such a method as an extremely promising therapy for this delicate issue in men into practical medicine. The practical value of the study lies in the systematisation of information on different sources of mesenchymal stem cells for treating erectile dysfunction.

## INTRODUCTION

Erectile dysfunction (ED) is a urological issue in men, affecting more than half of all men globally. It is known that the presence of diabetes significantly impairs bodily functions, and conditions such as diabetic neuropathy and angiopathy can lead to ED (1, 2). According to modern medical science, new treatment methods are needed for this diagnosis that could replace the not always effective use of symptomatic drugs. Therefore, mesenchymal stem cells, which can differentiate into various cell types, could help maintain the condition of male genital tissues in a normal state, even in the presence of diabetes (3). According to G.M. Irwin (4), who provided data from the IV International Consultation on Sexual Medicine, ED is defined as the periodic or constant inability to achieve or maintain an erection of the penis sufficient for sexual satisfaction. Currently, this pathology is a common problem, with up to 30 million men suffering from it annually in the United States alone.

Extensive epidemiological studies, such as the Massachusetts Male Aging Study (MMAS) and the European Male Aging Study (EMAS), have shown that men over 40 years old are in the main risk group for the disease; furthermore, the prevalence of the pathology increases by approximately 30% each year compared to previous figures (5). The last extensive study on erectile dysfunction in the Republic of Kazakhstan was conducted by Alchinbayev et al. (6) in 2014, and even at that time, statistical indicators pointed to a significant prevalence of nosology. According to the study, among 1550 men aged 21-79, erectile dysfunction was diagnosed in 52.3% of men. Based on the ageing trend of populations and the development of medicine, contemporary scientists believe that the prevalence of the disease by 2025 will exceed 60% of the male population; however, these data require further research (7).

Under physiological conditions, an erection is achieved through reflex and psychogenic pathways. Reflexive erection occurs through direct stimulation of the penis, which has significant innervation, while psychogenic erection is achieved through erotic and emotional stimuli directly dependent on the limbic system (8). It was previously believed that the dysfunction was solely due to psychological reasons; however, the scientific community now provides evidence of neurological causes of the disease. In particular, the development of various neuropathies and angiopathies has a statistically significant correlation with the occurrence of erectile dysfunction. Among them, the aetiology related to diabetes mellitus (DM) holds a special place. It has been established that the prevalence of the disease in patients with diabetes significantly exceeds that in other patients and ranges from 19% to 86.3%, according to various studies (9). Given that the International Diabetes Federation reports nearly 537 million people with diabetes worldwide, treating erectile dysfunction in these individuals becomes a serious problem (10).

Wang et al. (11) suggest that while conventional diabetes treatments mainly focus on preventing potential potency issues in patients, they often fall short in providing effective treatment. However, they highlight a promising alternative involving the use of mesenchymal stem cells administered through intracavernous injections. According to Feng et al. (12), this approach shows promise in alleviating erectile dysfunction symptoms by targeting ferroptosis in cells. Similarly, Xiong et al. (13) emphasize the regenerative and anti-apoptotic potential of this treatment method. While stem cell therapy is acknowledged as a promising avenue, these studies also caution about its short-lived therapeutic effects and the potential for developing side effects. Therefore, further research is needed to optimize its efficacy and safety profile for clinical application in treating erectile dysfunction associated with diabetes.

The purpose of this study is to assess the therapeutic potential of intracavernous injections of mesenchymal stem cells compared to other treatment methods and to analyse the prospective effectiveness of their use in clinical practice.

## MATERIALS AND METHODS

A comparative review of literary sources was conducted following modern PRISMA recommendations (14). This approach helped avoid inaccuracies and contributed to a more detailed description of the research methodology. Various aspects, including the accuracy and reliability of results, ethics, and the acceptability of the mentioned information, were considered in determining the criteria for acceptability. To ensure high-quality research, a generalised algorithm was developed, including detailed instructions for conducting the study and analysing the results. For the literature search to obtain relevant studies, analysis and systematisation methods considered sources from the Web of Science, Scopus, PubMed, Elsevier, and Springer databases from May 23, 2023, to September 14, 2023. At the initial stage of literature collection, the search was limited to English, Spanish, French, Russian, and Ukrainian languages, which could be a reason for excluding relevant studies by other authors in foreign languages.

To include the publication in the list of elaborated ones, broad selection criteria were deliberately chosen to better cover the area under study. Publications exploring possible theories and mechanisms of action of mesenchymal stem cells in erectile dysfunction over the last 5-10 years were automatically considered. Extreme special attention was paid to original clinical studies, systematic reviews and meta-analyses that examined and analysed the effectiveness of this treatment method in comparison with other methods and the possibilities of its use in patients with diabetes mellitus. Publications with study design flaws or containing a large amount of promotional material compared to the scientific content were not considered for further analysis. This was done to avoid unreliable information or false conclusions. Such an approach increased the reliability of the results and enhanced their significance.

The main research models falling within the scope of further review and investigation, as mentioned earlier as inclusion criteria, were: studies that allocated participants into control and experimental groups, with a minimum number of >10 participants; systematic reviews and meta-analyses; studies conducted to confirm or refute the effectiveness of using mesenchymal stem cells to treat erectile dysfunction; scientific publications on researching the effectiveness of using mesenchymal stem cells for healing erectile dysfunction and the mechanism of their action in intracavernous injections.

The criteria for exclusion from scientific analysis included publications with questionable results, advertising publications, and personal opinions of the authors on the treatment of erectile dysfunction in patients with diabetes mellitus without any scientific justification. After searching the literature in databases by key words and inclusion/exclusion criteria, 116 papers were found for primary research. The following key words were used in the research methodology to search for literature sources: mesenchymal stem cells, erectile dysfunction, diabetes mellitus, systematic reviews, meta-analyses, clinical studies, treatment effectiveness, research models, control and experimental groups, inclusion criteria, exclusion criteria. During interactive meetings, the authors collectively analysed the expediency of a particular study to achieve their goals. As a result, 48 publications were included in the study as references.

## RESULTS AND DISCUSSION

Diabetic erectile dysfunction (DED) is a pathological condition associated with chronic complications such as endothelial dysfunction, smooth muscle atrophy, and nerve degradation. In addition, endocrine and iatrogenic factors related to the medication treatment of diabetes and the side effects of drugs play a significant role (15). The classical therapy for ED so far has been phosphodiesterase inhibitors (PDE5).

**Figure 1:**
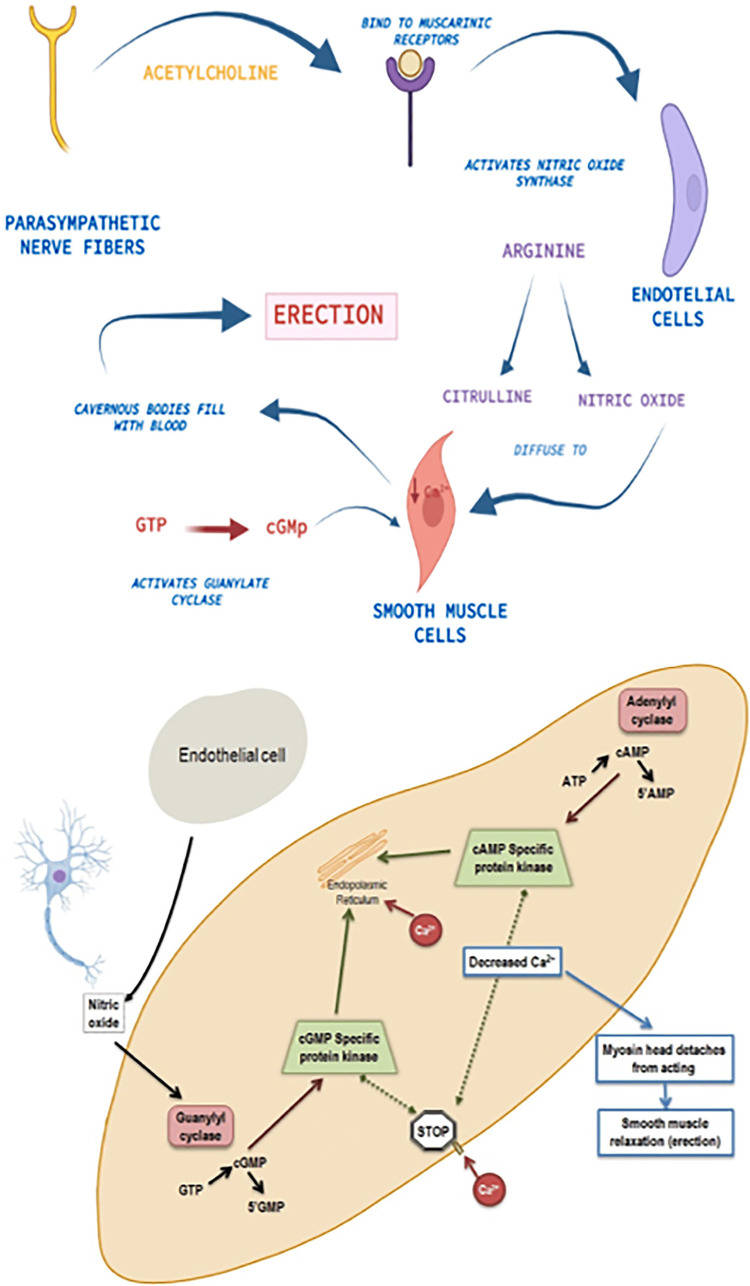
Physiology of erection and pathogenesis of erectile dysfunction.

Source: created by the authors.

However, according to G.P. Redrow (16), PDE5 inhibitors, such as sildenafil, have low efficacy in patients with DED. Therefore, stem cell therapy research has significant potential in this area. This is due to significant achievements in stem cell treatment in regenerative medicine and is explained by the potential regeneration of cavernous nerves and vessels affected by diabetes (17). The pathogenesis of the therapeutic effect of mesenchymal stem cells (MSCs) is based on their ability to replace damaged nerve or endothelial cells. Another hypothesis suggests that their use has a paracrine effect, regulating some of the key mediators involved in erection, such as calcium and nitric oxide concentrations. Perhaps the goal of MSC therapy is to regulate the endocrine aspects of ED (18, 19). Moreover, stem cells have anti-apoptotic and anti-fibrotic effects (Figure-1).

Several studies have examined the impact of different types of mesenchymal stem cells on the state of erection in men, among which positive effects were obtained from the treatment of HUCMSCs, BMSCs, and ADSCs (20). Starting in 2004, Bochinski et al. (21) first described the therapeutic benefits of treating ED as a secondary pathology due to various aetiologies. New data suggest that mesenchymal stem cells are effective in tissue engineering and disease rehabilitation (22). Several studies have shown for the first time that such stem cell therapy will be beneficial in treating ED. Thus, Ryu et al. (23), through experiments, created a rat model with diabetes and complications in the form of impotence, demonstrating that MSC transplantation improves erectile function in animals.

### Adipose Tissue-Derived Stem Cells

One of the most effective sources of MSCs is adipose tissue. ADSCs are essentially active cells involved in inhibiting apoptosis, revascularizing affected areas, and modulating immune processes. According to Mohseni et al. (24), adipose tissue-derived stem cells are adult and multipotent stem cells with the ability for self-renewal and differentiation. ADSCs are accessible through minimally invasive methods without causing harm to the host organism. In addition, successful allogeneic stem cell transplantation provides an understanding of the low immunogenicity of these cells.

Hou et al. (25) examined the use of adipose tissue-derived stem cells in rat models of ED. Meta-analysis results indicate that ADSCs treatment aids in regenerating damaged tissues and contributes to the restoration of sexual function. Significant attention has been given to the paracrine effect of stem cells, which release several substances that, in subgroup analysis, showed a greater effect. These substances include vascular endothelial growth factor (VEGF), brain-derived neurotrophic factor (NGF), and other factors. Positive data were particularly prominent in the subgroup of DED when ADSCs were used in combination with insulin therapy. Park et al. (26), in a meta-analysis, assessed the effects of adipose tissue-derived stem cells in rats with erectile dysfunction caused by cavernous nerve injury, which is one of the links in the pathogenesis of diabetes. The results demonstrated that ADSCs treatment improved erectile function based on indicators of penile hemodynamic and intracavernous pressure.

In a study by Garber and Carlos (27), adipose tissue-derived stem cells were injected into six patients diagnosed with DED. As a result of this treatment, 4 out of 6 patients experienced a positive effect. In the first month, spontaneous morning erections appeared, and the ability to engage in sexual intercourse persisted up to 12 months after SC injection. Haahr et al. (28) obtained similar results and concluded on the safety and effectiveness of this type of therapy, with the effect lasting for the following year after treatment. Protogerou et al. (29) made a discovery in their work on the technology of combined injection of stem cells and platelet-rich plasma lysate, which had no negative side effects for the next 6 months.

According to Chen et al. (30), conventional stem cell injections are less effective because adipose tissue cells are more differentiated; therefore, in the 2017 study, microtissues (MT) based on stem cells were used. Zhou et al. (31) confirmed the greater effectiveness of intracavernous injections of microtissues compared to single-cell ADSC injections in rats with DED. They determined that two weeks after the transplantation of labelled stem cells, a greater number of them was observed in the cavernous bodies after the injection of microtissues. This was also supported by other studies that investigated the paracrine effect of stem cells. The expression of vascular endothelium growth factor and brain neurotrophic factor was higher compared to MT with single-cell injections of SC. VEGF, as a pro-angiogenic factor, stimulates proliferation and has an anti-apoptotic effect, and a violation of the signalling pathway of this factor is associated with endothelial dysfunction in DM. The expression of NGF was more pronounced in the penises of diabetic rats after MT injection than in rats after ADSC injection (32).

Chen et al. (33) demonstrated the effectiveness of adipose-derived stem cell therapy for DED in rats, which may be attributed to paracrine effects. Erectile dysfunction is known to result from significant relaxation of the cavernous bodies of the male penis due to inadequate nitric oxide (NO) production. The endothelial fraction of NO plays a particularly important role, largely being one of the defining links in the pathogenesis of diabetic impotence. The same study showed that compared to BMSCs, ADSC therapy more effectively increased the expression of endothelial nitric oxide, and hematoxylin and eosin staining revealed a greater number of blood vessels in the cavernous bodies.

Studies have also confirmed the possibility of combining stem cell therapy with other cells. For instance, the effectiveness of combining ADSCs with endothelial precursor cells (EPCs) was analysed. EPCs are cells that can give rise to mature endothelial cells, which is essential for accelerating blood vessel regeneration. This property is necessary for men with diabetes, as it has been noted that the number of EPCs in diabetic men is significantly lower compared to healthy men. According to research data, injection with one type of cell has limited results (34). Yang et al. (35) confirmed the synergistic effect of ADSCs together with EPCs on endothelial function, significantly enhancing therapy results in a diabetic rat model with ED. As previously described, the VEGF signalling pathway plays a significant role in the pathogenesis of endothelial dysfunction. According to data, the endothelial growth factor is necessary for stimulating the differentiation of precursor cells into mature endothelial cells. This may explain the only partial effect of intracavernous EPC injections (36, 37). This also explains the positive and sustained effect of their synergistic action with stem cells.

### Bone Marrow-Derived Stem Cells (BMSCs)

BMSCs are a relatively common source of stem cells for therapeutic purposes. MicroRNAs play a significant role in the context of bone marrow cells. It has been established that they have a decisive role in the process of self-renewal and differentiation of stem cells into smooth muscle cells (SMCs), which are necessary to maintain an erection. This was confirmed in a study where the overexpression of these non-coding RNAs led to increased SMC expression (38). Liu et al. (39) found that BMSCs that overexpress microRNAs have a positive therapeutic effect through several mechanisms. Bone marrow cells, thanks to non-coding RNAs, increase the number of SMCs while acting on Krüppel-like factor 4 (KLF4), thereby reducing the production of collagen 1 and matrix metalloproteinase. In addition, BMSCs also reduce the phosphorylation of transforming growth factor-beta through its receptor TGFBR2. All the described links in the pathogenesis ultimately led to a significant improvement in erectile function in elderly rats.

Yiou et al. (40) disclosed the final results of phase II clinical trial (INSTIN study) that included 18 patients after radical prostatectomy and vasculogenic ED. Intracavernous injections of BMSCs were used in these patients, resulting in improved erectile function during a six-month follow-up with sustained results over a year. During the full follow-up period of 62.1 months, no side effects were reported; however, there was a slight deterioration in the initial therapeutic effect, which may lead to the repetition of the procedure. Al Demour et al. (41) conducted two clinical studies using autologous BMSCs in patients with diabetic ED. In the first phase, such injections were considered safe and effective, noting a considerable positive result in the International Index of Erectile Function (IIEF-15) and the Evaluation of Erectile Hardness Scale (EHS). In the second phase, two consecutive intracavernous injections of stem cells were used, which also had positive effects over the next 12 months.

### Umbilical Cord Mesenchymal Stem Cells

Human umbilical cord mesenchymal stem cells (HUCMSCs) are pluripotent and undifferentiated cells. Essentially, HUCMSCs are naive stem cells that can be harvested in large quantities. Compared to BMSCs and ADSCs, human umbilical cord cells have a relatively high positive effect in treating ED (42). Their use in therapy has some advantages compared to other sources of SCs. The first point is the non-invasive method of cell collection, significantly reducing the risk of side effects. Barrett et al. (43) demonstrated that these cells are more capable of proliferation and differentiation, as they can continue to grow steadily even after the tenth transplantation and passage while maintaining their effectiveness at the same dose. The tumorigenicity of these cells is relatively low, explained by clear information on the gene expression profile. Moreover, this also explains their high ability to differentiate and self-renew.

Cho et al. (44) found another explanation for why human umbilical cord cells can be an excellent option for ED therapy. This is primarily explained by the low likelihood of a “transplant-recipient organism” reaction since HUCMSCs are low-immunogenic. This is justified by extremely low expression of major histocompatibility complex class I and II molecules and certain ligands, such as CD40, CD80. Feng et al. (12) investigated the effect of umbilical cord mesenchymal stem cells on erectile function in rats with diabetic ED. Considerable positive therapeutic effects were noted without side effects, confirming the pathogenesis at the molecular level, demonstrating that HUCMSCs, by attenuating the ferroptosis of vascular smooth muscle cells, improve erection. Other sources of stem cells, such as bone marrow and adipose tissue, have negative aspects due to their high degree of stem cell differentiation, and lower stability since sildenafil is still needed for their treatment. Therefore, compared to them, umbilical cord cells had a high therapeutic result in rats with type 1 and 2 diabetes. Since HUCMSCs are of human origin, their ability to differentiate is somewhat limited, and it is more challenging for them to differentiate into rat penis stem cells to restore cavernous bodies, possibly resulting in only a small amount of colonization (36).

It has been shown that umbilical cord mesenchymal stem cells, despite their limitations, are capable of secreting several immunomodulatory factors and tissue regeneration factors, which have the property of reducing blood plasma glucose levels. Mesenchymal stem cells also reduce the degree of destruction of beta cells of the pancreas in patients with type 1 or type 2 diabetes, sometimes even improving their function. Their anti-inflammatory activity is also quite interesting, which, by the way, has been used in the treatment of low-grade chronic inflammatory processes in patients with type 2 diabetes (12). Thus, based on the above, it can be assumed that human umbilical cord mesenchymal stem cells, when intracavernously injected into patients with diabetes and complications in the form of erectile dysfunction, have a positive therapeutic effect in rat models of diabetic erectile dysfunction primarily due to the paracrine effect.

### Induced Mesenchymal Cells

Mesenchymal stem cells have low amplification capacity, causing them to age quickly, and their use in clinical practice is significantly limited. Therefore, modern scientists have started to explore new strategies, namely the invention of induced pluripotent stem cells. Induced pluripotent stem cells (iPSC) are stem cells synthesised through the reprogramming of somatic cells (45). These cells can also serve as a source of induced mesenchymal stem cells (iMSC), providing a new source of mesenchymal cells. Chen et al. (46) first described the use of iMSC in rats with ED. The therapeutic effect of these cells on the hemodynamic characteristics of the penis was evaluated. The effects observed were comparable to those of successful treatment with ADSC. When analysing the expression of indicators such as vWF, eNOS, SMA, and Desmin, which characterise endothelial dysfunction and the state of smooth muscle, it was determined that iMSC injections are not inferior in efficacy to other types of stem cells.

In addition, induced mesenchymal cells slightly increase the expression of nitric oxide, contributing to an increased sensitivity of receptors to sildenafil. The mechanism of the positive therapeutic effect of intracavernous iMSC injections is primarily due not to the cells themselves, as their concentration in the cavernous bodies significantly decreases three days after administration according to this study. However, their effectiveness is mainly associated with a paracrine effect and stimulation of the synthesis of various mediators by the host organism’s cells. The invention of a new stem cell induction technology and the development of iMSC allow the use of such therapy as a new strategy for treatment. It is evident that conventional sources of mesenchymal stem cells have certain negative aspects, namely ageing ability and low expansion property (47-49).

Nevertheless, induced MSCs have advantages in these problematic areas, making them a better vector for ED treatment. Firstly, iPSCs can be created from ordinary somatic cells, such as skin cells, making them obtainable non-invasively and with much fewer side effects. Secondly, these cells, unlike traditional MSCs, have the ability to expand their own population more than 120 times without ageing. Thirdly, induced mesenchymal stem cells are more homogeneous, as they originate from a single source of iPSCs (46-48).

### Ethical Constraints and Issues

ED is a urological problem that imposes a heavy psychological burden on the nervous system of men diagnosed with it. It affects overall well-being, self-esteem, and the social aspect of life, as sexual ability is an important part of health. In general, the analysis of treating diabetic erectile dysfunction with experimental stem cell therapy of various origins has shown significant and promising results in improving erectile function and erection in men with type 1 or type 2 diabetes, without the need for constant sildenafil use. However, despite most studies reporting positive results and the absence of side effects, there is still no general standardisation for the collection and use of specific types of stem cells. This complicates the use of stem cell treatment as a method in clinical practice. That is why stem cell therapy is an experimental method. Due to its experimental nature, lacking standardisation is observed in the production, harvesting, and use of stem cells. This leads to differences between laboratories and experimental centres working with stem cells. Such a difference can lead to variations and errors in experiments, including poor reproduction. That is why efforts are needed to standardise all stages of SC therapy (50-52).

Questions arise in stem cell therapy regarding the specific healing method and sources of stem cells. Despite limited human studies, the main sources for therapeutic use are bone marrow, adipose tissue, and umbilical/placental tissues. The primary delivery method is direct intracavernous injection, usually single-cell, with some studies using microtissues. Protocols involve isolating stem cells, expanding them in vitro, and delivering them into the body. Studies typically analyse therapeutic effectiveness, penile hemodynamic, and side effects.

## CONCLUSIONS

As a result of the literature analysis, it was established that intracavernous injections of mesenchymal stem cells for treating erectile dysfunction in patients with diabetes are an effective and promising method of therapy. The wide range of available preparations for use in the treatment of the disease allows for an individualised approach in the therapeutic management of each patient. However, to date, the most common are mesenchymal cells from the umbilical cord and bone marrow, which may primarily be associated with their general prevalence in treating other pathologies.

It was found that the main factors influencing the method were the regenerative and anti-apoptotic effects on the nervous and vascular structures within the penis. This compensates for potential damage in the capillary and venous plexuses of the organ due to hyperglycaemia and improves conductivity through sympathetic and parasympathetic nerve fibres, providing the reflex component of erection. In addition, this therapy method possesses antioxidant properties, reducing the amount of nitric oxide in cells and intercellular space, thereby preventing potential damage to nerve cells, pathways, vessels, and cavernous bodies. Due to such a comprehensive effect, one can conclude the effectiveness of intracavernous injections of mesenchymal stem cells for treating erectile dysfunction.

The effectiveness of this type of therapy is particularly observed in patients with diabetes, as they experience both neurogenic and angiogenic disorders. Furthermore, mesenchymal cells have the ability to produce immunomodulatory factors that, in turn, reduce the level of hyperglycaemia in the bloodstream. Another factor is the influence of mesenchymal cells on ferroptosis and the sensitivity of receptors to other drugs, such as sildenafil. However, the impact of mesenchymal stem cells is currently understudied and can be a promising area for further research.
